# Soil organic matter carbon chemistry signatures, hydrophobicity and humification index following land use change in temperate peat soils

**DOI:** 10.1016/j.heliyon.2023.e19347

**Published:** 2023-08-21

**Authors:** Apori Samuel Obeng, Julie Dunne, Michelle Giltrap, Furong Tian

**Affiliations:** a School of Food Science Environmental Health, Technological University Dublin, City Campus, Grangegorman, D07ADY7, Dublin, Ireland; b FOCAS Research Institute, Technological University Dublin, City Campus, Camden Row, D08C, CKP1, Dublin, Ireland

**Keywords:** FTIR, Hydrophilic, Hydrophobic, Humification index, Functional groups, Degree of degradation and peatland

## Abstract

Peatlands play a critical role in the global carbon cycle, storing large amounts of carbon because of a net imbalance between primary production and the microbial decomposition of the organic matter. Nevertheless, peatlands have historically been drained for energy sources (e.g. peat briquettes), forestry, or agriculture - practices that could affect the quality of the soil organic matter (SOM) composition, hydrophobicity and humification index. This study compared the effect of land use change on the quality and composition of peatland organic material in Co-Offaly, Ireland. Specifically, drained and grazing peat (grassland), drained and forest plantation peat (forest plantation), drained and industrial cutaway peat (cutaway bog) and an undrained actively accumulating bog (as a reference for natural peatland) were studied. Fourier-transform infrared spectroscopy (FTIR) was used to examine the organic matter quality, specifically the degree of decomposition (DDI), carbon chemistry signatures, hydrophobicity and humification index. The ratio of hydrophobic to hydrophilic group intensities was calculated as the SOM hydrophobicity. In general, there is greater variance in the carbon chemistry signature, such as aliphatic methyl and methylene, C=O stretching of amide groups, aromatic C=C, strong H-bond C=O of conjugated ketones and O–H deformation and C– O stretching of phenolics and secondary alcohols of the peat samples from industrial cutaway bog samples than in the grassland and forest plantation samples. The hydrophobicity and the aromaticity of the soil organic matter (SOM) are significantly impacted by land use changes, with a trend of order active bog > forest plantation > industrial cutaway bog > grassland. A comparison of the degree of decomposition index of the peat from active bog showed a more advanced state of peat degradation in grassland and industrial cutaway bog and, to a lesser extent, in forest plantation.

## Highlights

• Carbon chemistry signatures, hydrophobicity and humification index as affected by land use change in temperate peat soils were studied.
• Industrial cutaway bog and grassland exhibited higher decomposition of SOM than the forest plantation.
• Grassland (drained and grazing peat) exhibited the greatest humification index of SOM.
• The hydrophobicity and the aromaticity of the SOM follow the order active bog > forest plantation > industrial cutaway bog > grassland.

## 1 Introduction

Peatlands play a critical role in the global carbon cycle, as they store large amounts of carbon because of a net imbalance between primary production and the microbial decomposition of the organic matter [[1], [2], [3], [4]]. The presence of a high water level and the resulting anoxia, in combination with the poor quality of the litter (i. e high lignin content), and the limited availability of nutrients, inhibits the rapid rate of aerobic decomposition and further, much slower anaerobic decomposition [4,5]. Peatlands have historically been drained for energy, forestry, or agriculture, which could stimulate organic matter mineralisation [6,7], resulting in secondary peat decomposition (further breakdown of peat that has already undergone some degree of initial decomposition) processes brought on by chemical and physical transformation processes of the SOM composition [8,9]. Yet, the quality of the organic matter composition influences the amount of carbon locked up in the peat soil [9]. The SOM composition refers to the carbon-based structure of soil organic matter as well as the spatial arrangement of specific functional groups, such as carboxylic and hydroxylic groups, at the molecular level, and these functional groups dictate the soil organic matter's chemical reactivity and sorptivity [[10], [11], [12], [13], [14]].

The literature indicates that converting peatland to other land uses through drainage not only increases the soil acidity and decreases the organic carbon and total nitrogen but also negatively affects the hydrophobic functional component (C–H) of the organic matter, which in turn lowers the peat soil's hydrophobicity [[15], [16], [17], [18], [19]]. However, the increase in microbial activity and organic matter decomposition can be facilitated by reducing hydrophobicity in peat soil [20]. The presence of aliphatic C causes hydrophobicity of peat soil–H units on humic acid, which is naturally hydrophobic due to the waxy nature of its particles; non-polar groups (i.e. ethyl and methyl) and temporary aromatic compounds, which decrease the hydrophilicity; and absorbed hydrophobic substances, such as oil, fat and N-organic fractions on the particle surface [21,22]. As a result, the aliphatic C–H unit to the aromatic organic compound (C=O) ratio can be used to describe how water resistant the soil is and how much SOM degrades by microorganisms, as well as allow comparison in SOM composition between various peatland use changes [12,23,24]. Additionally, the hydrophilic constituents of the SOM increase the organic matter wettability, while hydrophobic constituents repel [17,19,25,26].

Fourier-transform infrared (FTIR) spectroscopy is a cost- and time-efficient spectroscopic tool capable of differentiating soil organic matter compositions as affected by peatland use changes [10,27,28]. FTIR is based on the absorption of infrared light, which results in a unique spectral fingerprint that can be used to describe the chemical composition and structural properties of SOM, such as carbohydrates, lignin, cellulose, fats and lipids and proteinaceous compounds, without the requirement of any extraction techniques [10,[29], [30], [31], [32]]. Niemeyer et al. [33] used transmission FTIR to analyse compost, humic acid, and peat samples and could identify relative changes in the profile of organic matter containing carbonyl bonds (C=O) during the humification process. Additionally, using FTIR, Artz et al. [12] studied the organic matter composition in peat samples at various stages of peatland regeneration from five European countries. FTIR has also been used to study the soil water repellence (soil hydrophobicity) and humification index of organic matter in mineral soil [19,[34], [35], [36]].

Despite the fact that drainage and land use change affect the soil water repellence (soil hydrophobicity) and humification index by altering the organic matter content, very few studies have investigated this phenomenon on peat soil relative to mineral soil [19,24,[35], [36], [37]]. For instance, Pärnpuu et al. [36] investigated the soil organic matter composition and soil water repellence/hydrophobicity of soil types in Estonia, but the study did not include soil from peatland. In a separate study, Heller et al. [28] assessed the impact of mire types and drainage intensity on the soil organic matter characterisation of temperate peatland. Still, the study focused only on SOM functional group and ignored some of the SOM characteristics, such as the degree of degradation and humification indices. However, grasping the mechanism of these issues is crucial for conserving the ecosystem function, such as peatlands' hydrological, ecological and biogeochemical functions [38]. Therefore, in this study, we compared the effect of land use changes on the quality and composition of peatland organic material in Co-Offaly, Ireland. Specifically, a drained and grazing peat (grassland), drained and forest plantation peat (forest plantation), drained and industrial cutaway peat (cutaway peat) and an actively accumulating peat as our reference (active bog). The hypothesis tested is that converting natural peatland (undrained) to other land use types negatively affects the carbon chemistry signatures of the organic matter, soil water repellence and humification index. The study will contribute to the existing literature on how human activity affects soil organic matter quality and our knowledge of creating efficient management plans for soil conservation and restoration.

## 2 Materials and methods

### 2.1 Study area

**Fig. 1:**
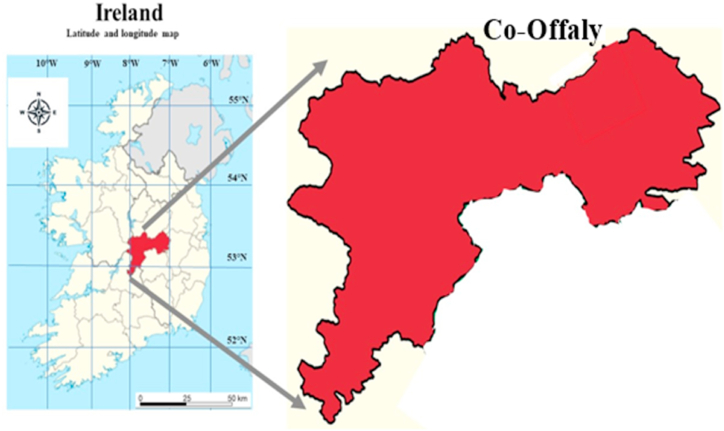
Location of the study area in the republic of Ireland.

Our study was conducted at Co-Offaly, located in the midlands of the Republic of Ireland (Fig. 1), in actively accumulating bog, industrial cutaway bog, grassland, and forest plantation (spruce). The county represents raised bogs well, a distinctive midlands landscape feature. However, it has been transformed from native to grassland, forest and industrial cutaway for energy sources and horticultural purposes since the 1980s by reducing the water level through drainage [39,40]. Across all the areas studied except for the actively accumulating bog, the drainage channels, which are still functioning, contain water with a depth of over 0.5 m, while their width exceeds 1 m and depth exceeds 1.5 m. The county's annual air temperature varies from a minimum of 5.7 °C to a maximum of 13.0 °C while the average precipitation is 819 mm [39].

This study used an active bog to reference natural peatland conditions, which remained unaltered. The purpose was to compare this reference with different land use types converted from the original natural peatland, including grassland, forestry plantation, and industrial cutaway bog. The active bog featured typical variations in its microtopography, such as hummocks and lawns, characterised by its vegetation. Dominant plant species were *Carex limosa* and *Trichophorum caespitosum*. *Sphagnum rubellum,* and *Sphagnum capillifolium,* were the species of the moss layer. The grassland primarily comprises perennial ryegrass, *Lolium perenne*, where the farmers regularly apply nitrogen sources such as slurry or inorganic fertilisers (at a rate of 40 kg/acre). The forestry peat soil was sampled from pine plantations, spruce plantations, ash plantations and sycamore plantations aged above ten years, while the peat samples of the cutaway bog were from a former raised bog that underwent industrial peat extraction primarily for electricity generation in a condensing power plant. The extraction process has been discontinued in these bogs, and a significant portion of the peat has been removed.

### 2.2 Soil sampling

The soil samples were collected from October 2021 to January 2022 across the Co-Offaly as we collected 135 peat soil samples from forest plantation (Twelve sites), grassland (twenty-five sites) and industrial cutaway bog (four sites) and undrained active bog (three sites). At each site, ten quadrants (0.5 m × 0.5 m) were positioned along a diagonal line of approximately 50 m × 50 m plots. Before collecting peat soil samples, the vegetation within each quadrant was trimmed down. The peat soil samples were obtained using a Russian peat corer (diameter, 15 cm), underneath with 20 cm intervals and then divided into two layers, 0–10 cm and 10–20 cm. Three composite peat soil samples (10 cores from the ten quadrants) from each site were put into plastic self-sealed bags. In the laboratory, 135 samples were oven dried (45 °C), crushed, and sieved through a 2-mm sieve before FTIR analysis.

### 2.3 ATR-FTIR spectroscopy

The dried and powdered peat from each location was analyzed using FTIR in attenuated total reflection (ATR) mode with a DTGS detector (PerkinElmer Spectrum One, PerkinElmer Inc., Waltham, MA, USA) to compare peat organic matter's carbon chemistry signatures among the various peatland use types. Anhydrous ethanol was used to clean the ATR crystal between samples. The instrument was calibrated against the background of the surrounding air between measurements to ensure that the results did not deviate due to changes in the lab's atmosphere. Peat samples were spectrally characterised with a scan range of 4000-650 cm^−1^ and a resolution of 4 cm^−1^. The OriginPro 2023 (OriginLab Corporation, Northampton, MA, USA) was used for processing spectra data to correct the baseline (connecting line method), smoothing (Savitzzky-Golay method) and normalisation. The peak intensity (by height) was recorded to assess the magnitude of infrared absorption of the selected peaks. The absorption bands recorded were assigned to their organic carbon assignment (Table 1).

**Table 1:** Peak positions in the FT-IR spectra reported in the literature and their proposed assignments [24,29,30,41,42].

Peak Name	Integration limits (cm^−1^)	Characterisation
1050	1070–1040	C–O Stretching and O–H deformation
1110	1116–1080	Secondary alcohol
1410	1420–1410	O–H deformation and C– O stretching of phenolics group
1520	1535–1500	Aromatic rings, amides II vibration
1610	1620–1600	Aromatic C = C stretch, strong H-bond C=O of conjugated ketones
1640	1660–1630	C=O stretching of amide groups
2921, 2983	3020–2800	Symmetric and asymmetric C single bond H stretching of CH_3_ and CH_2_ groups

### 2.4 Humification index

FTIR spectroscopy is used to determine the changes in humification that occur with depth based on the relative presence of resistant moieties, such as aromatic and phenolics compounds, compared to the abundance of labile fractions, such as carbohydrates [30]. The humification index can be determined by using the absorption peaks 1050, 1410 and 1520 cm^−1^ corresponding to C–O stretching of polysaccharides, O–H deformation and C– O stretching of phenolics and aromatic ring, respectively, which are the most indicate the organic matter structure [8,30]. As a result, the following equation was used to calculate the humification index:

(1) $$Humification\;index\;\left(a\right)=\frac{carboxylate\;structures\;\left(wavenumber\;at\;1410\;cm-1\right)}{polysaccharide\;content\;\left(Wavenumber\;at\;1050\;cm-1\right)}$$

(2) $$Humification\;index\;\left(b\right)=\frac{aromatic\;and\;phenolic\;compounds\;relative\;\left(Wavenumber\;at\;1520\;cm-1\right)}{polysaccharidride\;content\;\left(Wavenumber\;at\;1050\;cm-1\right)}$$

### 2.5 Hydrophobicity and aromaticity

The hydrophobic/hydrophilic group was calculated as the parameter of peat soil hydrophobicity or SOM water repellence [43]. The hydrophobic/hydrophilic signifies the ratio of (C–H) groups relative to those of the (C=O) group of the peat substrate. According to Heller et al. (2015), the presence of high hydrophobicity (indicated by the ratio of C–H to C=O bonds) suggests a certain level of protection for soil organic matter (SOM) against microbial degradation. Therefore, the hydrophobicity of the peat soil was estimated using the following equation.

(3) $$Hydrophobicity=\frac{Hydrophobic\;functional\;groups}{Hydrophilic\;functional\;groups}$$

where hydrophobic and hydrophilic functional groups were estimated using the peak height at (2983 + 2921 cm^−1^) and 1720 cm^−1^, respectively.

The aromaticity of the SOM was estimated by the ratio of aromatic (1610 + 1520 cm^−1^) to the aliphatic groups (2983 + 2921 cm^−1^).

### 2.6 Degrees of decomposition

The decomposition of SOM has traditionally been measured by the proportion of hydrophilic compounds to the combined aromatic and aliphatic compounds [36,44]. As a result, the peak intensity at 1720 cm^−1^, representing the hydrophilic components, was divided by the total of the peak height at 2983 + 2921 cm^−1^ to determine the degree of decomposition of peat organic matter.

### 2.7 Data analysis

The study's data were statistically analyzed using one-way ANOVA in OriginPro learning edition software. Post-hoc analysis (Bonferroni test) with α value of 0.05 was used to assess the significance of the data among the peatland use changes (grassland, industrial cutaway bog, and forest plantation). Correlation and Principal component analysis (PCA) were used to evaluate the interrelationships between the carbon chemistry signature, components, and characteristics of organic matter of peat soil following land use change.

## 3 Results

### 3.1 Carbon chemistry signatures of soil organic matter

**Fig. 2:**
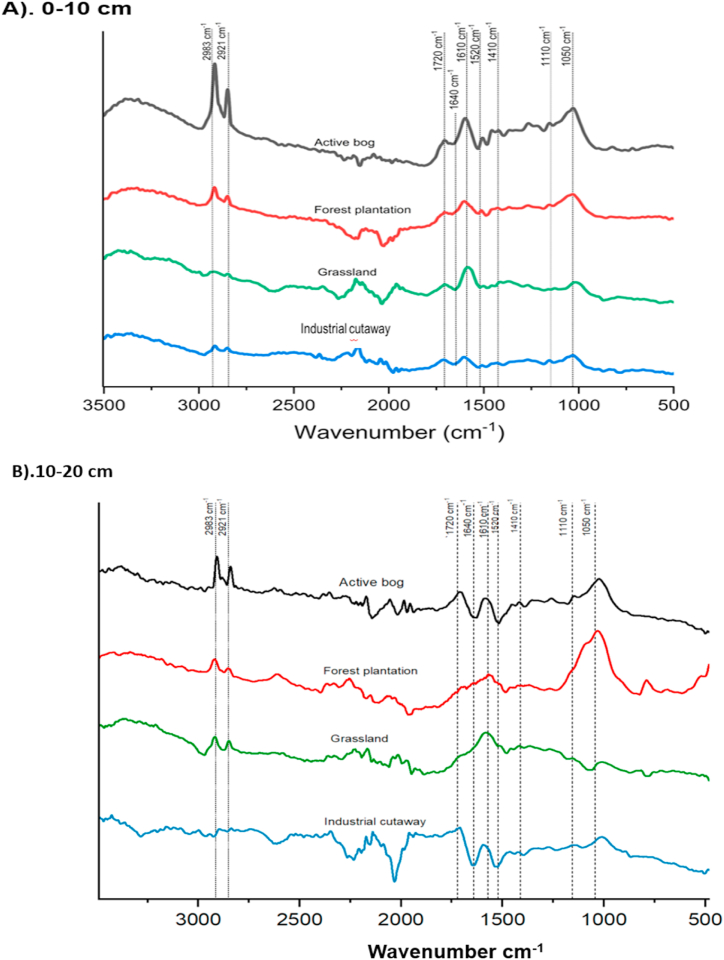
Typical FTIR spectra of 0–10 cm (A) and 10–20 cm (B) peat soil following land use change in temperate peat soils.

The Fourier Transform Infrared (FTIR) spectra of the peat soil under the land use type exhibited resemblances, as illustrated in Fig. 2. However, the distinction in carbon chemistry attributes of the peat soil derived relied on the analysis of peak intensity (specifically, the height of specific peaks). The distribution of the carbon chemistry signatures within SOM as a function of land use change is given in Table 2. The aliphatic methyl and methylene (peaks, 2921 and 2983) group did not differ significantly among the studied land use change for the two soil depths (0–10 and 10–20). However, In the 0–10 cm, the aliphatic methyl and methylene (peak, 2921) group was higher in grassland, whiles in the 10–20 cm depth, the forest plantation exhibited the highest aliphatic methyl and methylene group. Also, the aliphatic methyl and methylene group (peak, 2983) was higher in the active bog (reference sample) for 0–10 and 10–20 cm depth. In 0–10 cm, the C=O stretching of amide groups (peak, 1640) varies differently among the land use change. The active bog and the cutaway bog exhibited the highest and lowest C=O stretching of amide groups, respectively. The C=O stretching of amide groups did not differ significantly (P > 0.05) between the grassland and the forest plantation. In 10–20 cm, grassland exhibited the lowest C=O stretching of amide groups (Peak 1640). The C=O stretching of amide groups (peak, 1640) indicated by the forest plantation did not differ significantly from that of the cutaway bog. In the two soil depths (0–10 and 10–20 cm), the grassland exhibited the lowest aromatic C=C, strong H-bond C=O of conjugated ketones (peak, 1610) among the land use change (Forest plantation and cutaway). However, the forest plantation aromatic C=C, strong H-bond C=O of conjugated ketones (peak, 1610) was higher than cutaway bog but did not differ significantly (P > 0.05). The aromatic ring (peak, 1520) did not differ among the land use changes. The forest plantation exhibited a higher aromatic C=C ring than the cutaway and grassland. Also, the studied land use change showed decreased O–H deformation and C– O stretching of phenolics (peak, 1410) compared to the active bog. The O–H deformation and C– O stretching of phenolics was higher in the forest plantation than in the grassland and the cutaway bog in 0–10 and 10–20 cm depths. The grassland exhibited the lowest secondary alcohols (peak, 1110) and the C–O stretching of polysaccharides (peak, 1050) than when natural peatland is being converted to forest plantation (Table 2).

**Table 2:** Peak intensity (x10^2^) determined by ATP-FTIR of peat soil following land use change.

Peak number (cm^−1^)	Active bog	Cutaway bog	Forest Plantation	Grassland	*Significant*^*X*^	*F*^*Y*^
***0-*10 cm**						
1050	4.655 ± 0.021^a^	3.224 ± 0.001^ab^	1.956 ± 0.014^bc^	0.997 ± 0.011^c^	***	8.024
1110	4.006 ± 0.029^a^	0.985 ± 0.006^b^	1.924 ± 0.017^b^	0.834 ± 0.005^b^	***	8.198
1410	4.214 ± 0.017^a^	0.931 ± 0.004^b^	1.947 ± 0.016^b^	1.075 ± 0.010^b^	**	8.387
1520	2.178 ± 0.014^a^	0.845 ± 0.001^a^	1.188 ± 0.010^a^	1.034 ± 0.009^a^	NS	1.759
1610	3.956 ± 0.013^a^	1.189 ± 0.005^b^	1.755 ± 0.014^b^	1.132 ± 0.008^b^	***	8.482
1640	4.326 ± 0.015^a^	0.705 ± 0.005^b^	1.764 ± 0.015^b^	1.029 ± 0.009^b^	**	10.388
2921	1.062 ± 0.008^a^	0.737 ± 0.006^a^	1.072 ± 0.0166^a^	1.161 ± 0.012^a^	NS	0.1382
2983	1.497 ± 0.019^a^	1.478 ± 0.015^a^	0.991 ± 0.017^a^	1.298 ± 0.013^a^	NS	0.200
**10-20 cm**						
1050	5.785 ± 0.025^a^	3.945 ± 0.016^a^	2.143 ± 0.013^ab^	1.171 ± 0.009^b^	***	8.681
1110	6.953 ± 0.026^a^	3.455 ± 0.033^b^	1.718 ± 0.010^bc^	0.916 ± 0.006^c^	***	22.309
1410	4.206 ± 0.029^a^	2.926 ± 0.009^ab^	1.432 ± 0.013^b^	1.028 ± 0.010^b^	**	6.268
1520	4.717 ± 0.006^a^	3.531 ± 0.01^ab^	1.698 ± 0.014^ab^	0.015 ± 0.019^b^	**	4.472
1610	3.886 ± 0.012^a^	1.693 ± 0.012^ab^	1.974 ± 0.017^ab^	0.992 ± 0.006^b^	**	5.447
1640	4.627 ± 0.013^a^	3.22 ± 0.023^ab^	1.736 ± 0.013^bc^	0.963 ± 0.005^c^	***	14.761
2921	0.55 ± 0.006^a^	1.091 ± 0.012^a^	1.644 ± 0.012^a^	1.347 ± 0.018^a^	NS	0.452
2983	1.798 ± 0.022^a^	1.64 ± 0.014^a^	1.341 ± 0.017^a^	1.18 ± 0.012^a^	NS	0.256

F^*Y*^ and Significant^*X*^ effects were obtained from a one-way analysis of variance. NS= Not significant; Means followed by the same letter in each row are not significantly different at *p ≤ 0.05; **p ≤ 0.01***p ≤ 0.001 using Bonferroni.

### 3.2 SOM components of peat soil following land use changes

In the two soil depths (0–10 and 10–20 cm), converting natural peatland to the studied land use did not differ significantly (P > 0.05). In 0–10 cm depth, the hydrophilic ranged from 0.238 to 1.881, with the lowest and the highest value exhibited by the active and the cutaway bog, respectively (Table 3). In 10–20 cm, the forest plantation, cutaway, and grassland exhibited 0.396, 0.372 and 1.51, respectively. In the 0–10 cm, the hydrophobic components of the SOM following land use change vary from 2.063 to 2.559 such that the forest plantation and the active bog exhibited the lowest and the highest value, respectively, whiles in the 10–20 cm, the hydrophobic ranged from 2.349 to 2.985 with the lowest and the highest value of the range exhibited by the active and the forest plantation (Table 3). The aromatic component of the SOM varies significantly (P < 0.05) among the studied land use. In 0–10 cm, the aromatic component of the SOM ranged from 2.034 to 6.134, with the lowest and the highest end of the range exhibited by the cutaway and the active bog, respectively. The forest plantation recorded a higher aromatic component of the SOM than the grassland but did not differ significantly (P > 0.05). In the 10–20 cm, the grassland exhibited the lowest aromatic component of the SOM among the studied land use (Table 3).

**Table 3:** Organic matter components of peat soil following land use change.

Land use change	SOM components (peak intensity x10^2^)		
	Hydrophilic	Hydrophobic	Aromatic
**0-10 cm**			
Active bog	0.238 ± 0.002^a^	2.559 ± 0.028^a^	6.134 ± 0.013^a^
Cutaway bog	1.881 ± 0.011^a^	2.214 ± 0.016^a^	2.034 ± 0.005^a^
Forest Plantation	0.399 ± 0.005^a^	2.063 ± 0.033^a^	2.942 ± 0.021^a^
Grassland	1.089 ± 0.012^a^	2.467 ± 0.024^a^	2.165 ± 0.018^a^
*Significant*^*X*^	NS	NS	***
*F*^*Y*^	3.60	0.07	6.23
**10-20 cm**			
Active bog	0.862 ± 0.012^a^	2.349 ± 0.021^a^	8.602 ± 0.029^a^
Cutaway bog	0.372 ± 0.003^a^	2.732 ± 0.008^a^	5.224 ± 0.101^ab^
Forest Plantation	0.396 ± 0.021^a^	2.985 ± 0.027^a^	3.673 ± 0.031^b^
Grassland	1.51 ± 0.022^a^	2.527 ± 0.025^a^	2.499 ± 0.021^b^
*Significant*^*X*^	NS	NS	***
*F*^*Y*^	3.75	1.012	6.752

F^*Y*^ and Significant^*X*^ effects were obtained from a one-way analysis of variance. NS= Not significant; Means followed by the same letter in each column are not significantly different at *p ≤ 0.05; **p ≤ 0.01***p ≤ 0.001 using Bonferroni.

### 3.3 Organic matter characteristics of peat soil following land use changes

In 0–10 cm, the hydrophobicity differs significantly (P 0.05) among the studied land use (Table 4). The hydrophobicity ranged from 1.246 at the cutaway bog to 9.448 at the active bog. The forest plantation observed a higher hydrophobicity than the grassland, but it did not differ significantly (P > 0.05) (Table 4). In 10–20 cm, the hydrophobicity did not differ significantly (P > 0.05) among the studied land use. The hydrophobicity ranged from 5.09 to 7.30, with the lowest and the highest value exhibited by the grassland and the active bog, respectively (Table 4). In the two depths (0–10 and 10–20 cm), the aromaticity differs significantly (P < 0.05) among the studied land use. In the 0–10 cm, the aromaticity ranged from 1.47 to 5.10, with the lowest and the highest observed by grassland and the active bog, respectively. The aromaticity of the cutaway bog did not differ significantly (P > 0.05) from that of the forest plantation. In the 10–20 cm, grassland observed the lowest aromaticity compared to the other studied land use (forest plantation, cutaway bog, and active bog). The humification indices (A and B) did not differ significantly (P > 0.05) among the studied land. In the two depths (0–10 cm and 10–20 cm), the grassland exhibited the highest humification indices (A and B) (Table 4).

**Table 4:** Organic matter characteristics of peat soil following land use change.

Land use change	Hydrophobicity	Aromaticity	Humification Index (HIA)	Humification index (HIB)
**0-10 cm**				
Active bog	9.448 ± 4.885^a^	5.104 ± 2.655^a^	0.54 ± 0.139^a^	0.561 ± 0.281^a^
Cutaway bog	1.246 ± 0.412^b^	1.761 ± 1.682^ab^	0.288 ± 0.005^a^	0.263 ± 0.023^a^
Forest Plantation	4.525 ± 2.486^b^	3.017 ± 2.640^ab^	0.780 ± 0.474^a^	0.845 ± 0.147^a^
Grassland	2.756 ± 1.626^b^	1.472 ± 1.654^b^	1.889 ± 0.492^a^	1.758 ± 0.293^a^
*Significant*^*X*^	***	**	NS	NS
*F*^*Y*^	12.540	4.427	1.871	1.21
**10-20 cm**				
Active bog	7.304 ± 1.819^a^	10.453 ± 5.413^a^	0.861 ± 0.069^a^	0.865 ± 0.152^a^
Cutaway bog	5.697 ± 1.314^a^	2.028 ± 0.712^ab^	0.841 ± 0.199^a^	0.953 ± 0.612^a^
Forest Plantation	6.946 ± 2.577^a^	3.139 ± 2.314^ab^	0.912 ± 0.208^a^	0.947 ± 0.1612^a^
Grassland	5.091 ± 2.012^a^	1.938 ± 1.012^b^	1.423 ± 0.272^a^	1.609 ± 0.338^a^
*Significant*^*X*^	NS	**	NS	NS
*F*^*y*^	12.53	3.510	1.461	1.25

Humification index using the ratio of (HIA) carboxylic/carboxylate structures relative to polysaccharide content and (HIB) aromatic and phenolic compounds relative to polysaccharide content. F^*Y*^ and Significant^*X*^ effects were obtained from a one-way analysis of variance. NS= Not significant; Means followed by the same letter in each column are not significantly different at *p ≤ 0.05; **p ≤ 0.01***p ≤ 0.001 using Bonferroni.

### 3.4 Degree of decomposition

**Fig. 3:**
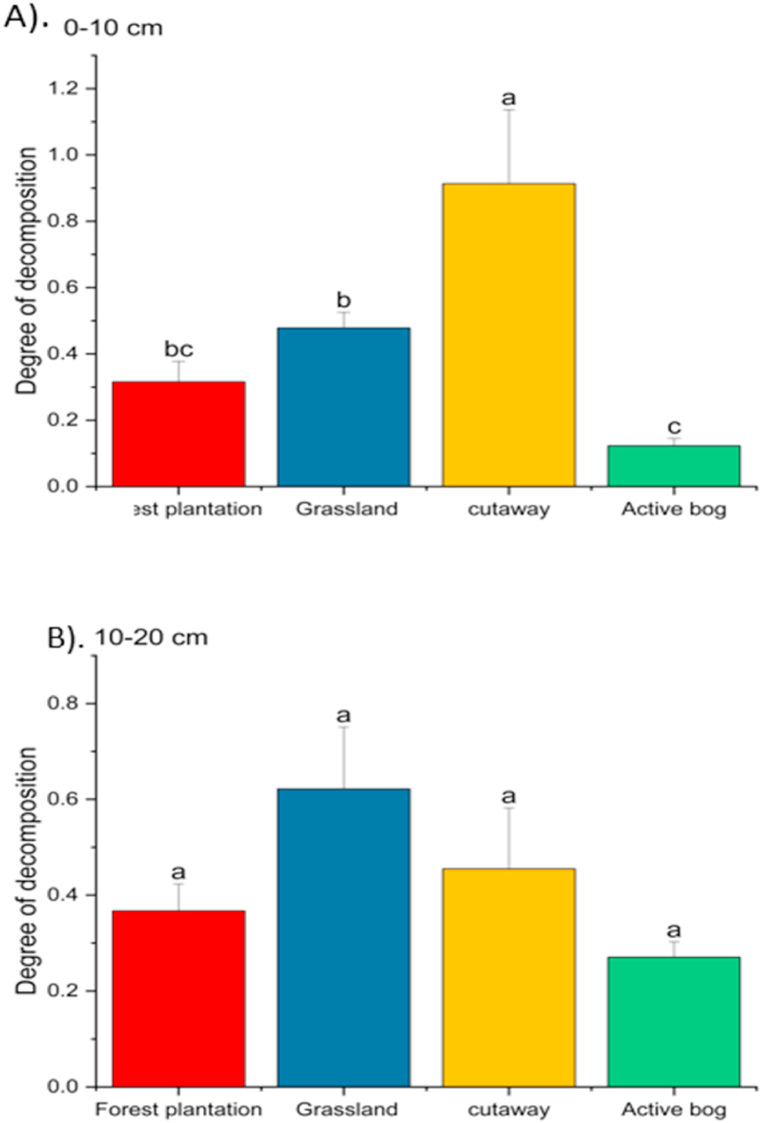
Degree of Decomposition of 0–10 cm (A) and 10–20 cm (B) peat soil *following land use change. The same letters on the bars are not significantly different at P 0.0 5 using Bonferroni at the 0.05 significance level; Error bars represent standard deviation of the means.*

The cutaway bog exhibited the highest DDI in 0–10 cm depth. The DDI recorded by the grassland was higher than the forest but did not differ significantly (P > 0.05) (Fig. 3A). The DDI in 10–20 cm depth did not vary significantly among the studied land use (P > 0.05). However, the highest DDI was exhibited by grassland, followed by the cutaway and the forest plantation (Fig. 3B).

### 3.5 Multivariate analyses-Pearson moment and principal component analysis of carbon chemistry signatures, components, and characteristics of organic matter of peat soil following land use change

**Fig. 4:**
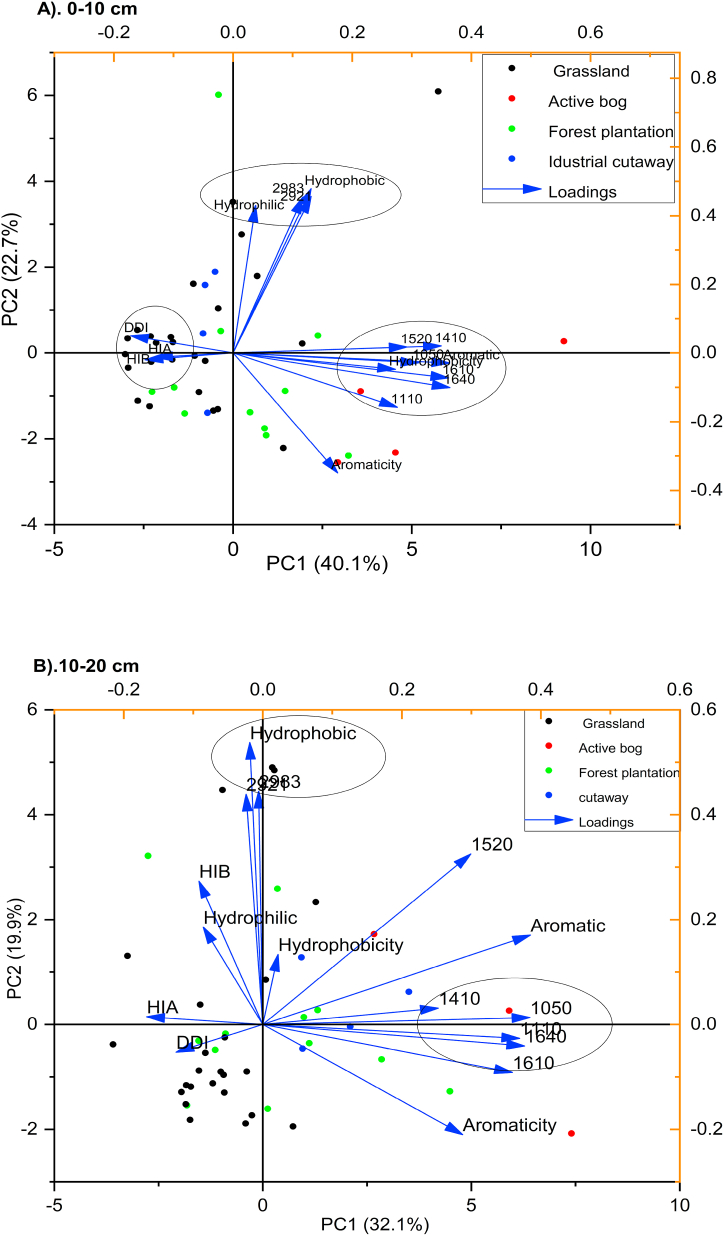
Biplots of 0–10 cm (A) and 10–20 cm (B) peat soil organic matter quality indicators affected by land use change; HIA-Humification index using the ratio of carboxylic/carboxylate structures relative to polysaccharides content; HIB-Humification index using the ratio of aromatic and phenolic compounds relative to polysaccharides content.

Principal component analysis (PCA) is a multivariate technique that can reduce the number of variables in a dataset by retaining the component that makes up most of the variation in the data and showing correlations and trends in a graph. Therefore, Principal Component Analysis with eigenvalues greater than one was performed on carbon chemistry signatures, components, and characteristics of organic matter of peat soil. The loadings from the first two PCAs of the data of 0–10 cm and 10–20 cm depth are shown in Fig. 4A and B, respectively. In 0–10 cm depth, the first principal component (PC1) explained 40.1% variance, with the variation in the data described by the carbon signature, aromaticity, hydrophobic, hydrophilic, and aliphatic methyl and methylene group (peaks 2983) and therefore demonstrates a significant degree of variation among grassland, forest plantation and cutaway bog compared to the active bog (Fig. 4A). The association between hydrophobic components and the aliphatic methyl and methylene group (peaks 2983) may give a clue that the increase or decrease of the aliphatic methyl and methylene group (peaks 2983) of organic matter may have a direct impact on the hydrophobic component (Fig. 4A). The second principal component (PC2) is explained 22. 7% of the variance, with major contributions provided by DDI, HIA and HIB within the grassland and the forest plantation area (Fig. 4B). In the 10–20 cm depth, PC1 and PC2 explained 32.1% and 19.9% of the variance, respectively. The PC1 loaded hydrophobicity, aromatic, aromaticity, and the carbon chemistry signature; however, most of the organic carbon signatures and the aromaticity occurred at the negative compartment of the PC1. The variation in the data in the PC2 was contributed by HIB, DD1, HIB, hydrophilic and hydrophobic (Fig. 4B).

The Pearson correlation was used to identify the interrelationship between the carbon chemistry signatures, organic matter components, characteristics, and degree of decomposition of the peat soil. In 0–10 cm, the organic matter components of the peat soil (aromatic, hydrophobic and hydrophilic) nearly exhibited a positive correlation (r > 0.183) with all the carbon chemistry signatures of the peat soil exception of O–H deformation and C– O stretching of phenolics (peak, 110) which exhibited a weak negative correlation with hydrophilic and hydrophobic functional groups (Table 5). Although most carbon chemistry signatures correlated positively with the peat soil's hydrophilic and hydrophobic components, the correlations were insignificant (P > 0.05). The only significant correlation was observed between the aliphatic methyl and methylene group (peaks 2921 and 2983) and the hydrophilic and hydrophobic components of the peat soil (Table 5). However, the aromatic component of the peat soil exhibited a significant correlation with nearly all the carbon chemistry signatures except for the aliphatic methyl and methylene group. The aromaticity nearly exhibited a significant positive correlation (r > 0.2983 at 0.05 level of significance) with all the carbon chemistry signatures found in the peat soil with the exclusion of secondary alcohols (peak, 1110). Also, the aromaticity showed a significant weak negative correlation (r > −0.362 at 0.05 level of significance) with aliphatic methyl and methylene group (peaks 2921 and 2983). The hydrophobicity nearly showed a significant positive correlation (r > 0.254 at 0.05 level of significance) with all the carbon chemistry signatures found in the peat soil and the aromaticity (Table 5). The degree of decomposition of the peat soil exhibited a significant negative correlation with the C=O stretching of amide groups (peak, 1640) and hydrophilic. The humification indices (HIA and HIB) exhibited a negative correlation (r > −0.472 at 0.05 level of significance) with the C–O stretching of polysaccharides (peak, 1050). In the 10–20 cm, the aromatic exhibited a significant positive correlation with aromaticity (r > 0.572 at 0.05 level of significance) and all the identified carbon chemistry signatures (r > 0.131 at 0.05 level of significance) except for aliphatic methyl and a methylene group (peaks, 2921 and 2983) (Table 5). The hydrophobic showed a significant positive correlation (r > 0.302 at 0.05 level of significance) with HIB, hydrophobic, aromaticity, aliphatic methyl, and a methylene group (peaks, 2921 and 2983) and the aromatic ring (peak, 1520). The hydrophilic exhibited a significant positive correlation with the aliphatic methyl and methylene group (peak, 2921), degree of decomposition and hydrophobic. Unlike the 0–10 cm, the hydrophobicity of the peat soil exhibited a non-significant positive correlation with all the carbon chemistry signatures (Table 5).

**Table 5:** Pearson correlation matrix of component analysis of carbon chemistry signatures, components, and characteristics of organic matter of peat soil.

Peak number (cm^−1^)	Aromatic	Aromaticity	Hydrophobic	Hydrophilic	Hydrophobicity	DDI	HIA	HIB
**0-10 cm**								
1610	0.914***	0.438**	0.144^ns^	0.076^ns^	0.533**	−0.251^ns^	−0.224^ns^	−0.249^ns^
1520	0.847**	0.370**	0.225^ns^	0.268^ns^	0.254^ns^	−0.239^ns^	−0.087^ns^	−0.047^ns^
1410	0.892***	0.380**	0.184^ns^	0.064^ns^	0.543***	−0.290^ns^	−0.172^ns^	−0.216^ns^
1050	0.636***	0.380**	0.171^ns^	0.123^ns^	0.479***	−0.188^ns^	−0.472**	−0.551**
1640	0.907**	0.493***	0.118^ns^	−0.018^ns^	0.581**	−0.336**	−0.187^ns^	−0.196^ns^
1110	0.593***	0.293^ns^	−0.016^ns^	−0.260^ns^	0.591***	−0.214^ns^	−0.231^ns^	−0.231^ns^
2983	0.183^ns^	−0.431**	0.959*	0.624***	0.305***	−0.192^ns^	−0.116^ns^	−0.170^ns^
2921	0.204^ns^	−0.362**	0.943***	0.651***	0.203^ns^	−0.154^ns^	−0.079^ns^	−0.077^ns^
Aromatic		0.462***	0.202^ns^	0.179^ns^	0.466**	−0.278^ns^	−0.186^ns^	−0.183^ns^
Aromaticity	0.462***		−0.420**	−0.458**	0.321*	−0.260^ns^	−0.160^ns^	−0.156^ns^
Hydrophobic	0.202^ns^	−0.420***		0.669***	−0.271^ns^	−0.184^ns^	−0.104^ns^	−0.133^ns^
Hydrophilic	0.179^ns^	−0.458**	0.669***		−0.328***	0.387**	−0.055^ns^	−0.048^ns^
Hydrophobicity	0.466*	−0.321***	0.271^ns^	−0.328***		−0.719**	−0.086^ns^	−0.167^ns^
DDI	−0.278^ns^	−0.260^ns^	−0.184^ns^	0.387**	−0.719**		0.002^ns^	0.050^ns^
HIA	−0.062^ns^	−0.154^ns^	−0.168^ns^	−0.105^ns^	−0.142^ns^	0.115^ns^		0.930***
HIB	−0.186^ns^	−0.162^ns^	−0.130^ns^	−0.045^ns^	−0.171^ns^	0.054^ns^	0.930**	
**10-20 cm**								
1610	0.794***	0.764***	−0.170^ns^	−0.226^ns^	0.017^ns^	−0.172^ns^	−0.190^ns^	−0.138^ns^
1520	0.884***	0.267^ns^	0.427***	0.035^ns^	0.095^ns^	−0.200^ns^	−0.262^ns^	0.358***
1410	0.432**	0.105^ns^	−0.002^ns^	−0.184^ns^	0.138^ns^	−0.193^ns^	0.015^ns^	−0.210^ns^
1050	0.417**	0.037^ns^	0.038^ns^	−0.288^ns^	0.247^ns^	−0.292^ns^	−0.336**	−0.329**
1640	0.659***	0.425***	−0.120^ns^	−0.226^ns^	0.053^ns^	−0.197^ns^	−0.215	−0.186^ns^
1110	0.598***	0.432**	−0.073^ns^	−0.187^ns^	0.095^ns^	−0.170^ns^	−0.212	−0.226^ns^
2983	0.198^ns^	−0.313**	0.805***	0.043^ns^	0.228^ns^	−0.135^ns^	0.122^ns^	0.333***
2921	0.131^ns^	−0.302**	0.836**	0.438**	0.165^ns^	−0.116^ns^	−0.180^ns^	0.179^ns^
Aromatic		0.572***	0.199	−0.093^ns^	0.073^ns^	−0.223^ns^	−0.273^ns^	0.169^ns^
Aromaticity	0.572***		−0.374**	−0.219^ns^	−0.155*	−0.095^ns^	−0.174^ns^	−0.087 ^ms^
Hydrophobic	0.199^ns^	−0.374***		0.302***	0.238^ns^	−0.153^ns^	−0.043^ns^	0.308**
Hydrophilic	−0.093^ns^	−0.219^ns^	0.302***		−0.395^ns^	0.737***	0.061^ns^	0.190^ns^
Hydrophobicity	0.073^ns^	−0.155*	0.238^ns^	−0.395**		−0.394**	−0.079^ns^	−0.047^ns^
DDI	−0.223^ns^	−0.095^ns^	−0.153^ns^	0.737***	−0.394***		0.217^ns^	0.171^ns^
HI-A	−0.273^ns^	−0.174^ns^	−0.043^ns^	0.061^ns^	−0.079^ns^	0.217^ns^		0.591**
HI–B	0.169^ns^	−0.087^ns^	0.308***	0.190^ns^	−0.047^ns^	0.171^ns^	0.591***	

DDI = Degree of decomposition; HIA-humification index using the ratio of carboxylic/carboxylate structures relative to polysaccharides content; HIB- humification index using the ratio of aromatic and phenolic compounds relative to polysaccharides content; Significant at *p ≤ 0.05; **p ≤ 0.01***p ≤ 0.001 using Bonferroni; ^ns^ = non-significant.

## 4 Discussion

Our study indicated that converting natural peatland to the studied land use through drainage decreased the carbon chemistry signatures of the SOM except for the aliphatic methyl and methylene group occurring at the 10–20 cm depth, which agrees with the results observed by Negassa et al. [45]. The highest aliphatic methyl and methylene groups observed in the active bog may be attributed to changes in the molecular structure of the alkyl groups, indicating a transformation of the organic matter in the active bog from simpler forms to more resistant aliphatic methyl and methylene groups occurring in the 10–20 cm depth [46,47].

The grassland and the forest plantation exhibited higher aliphatic methyl and methylene groups than the cutaway bog suggesting an increase in the decomposition of more easily degradable carbohydrates in the forest and the grassland peat soil. Grassland observed the lowest NH_2_ and NH-bending in primary and secondary amides, resulting in the decline of the nitrogen (N) content from the peatland system, causing nitrogen gas emission, as reported by Olk et al. [48]. The decreased aromatic C=C in the studied land use implies the decomposition of the lignin content and the polyphenolics compounds of the organic matter of the peat soil [31]. Therefore, it can be substantiated that land use changes contribute to the degradation of the organic matter quality of the peat through the decomposition of the phenolic component of peat since the presence of the phenolics suppresses bacterial and fungal decomposition and extracellular enzyme activity [31]. Freeman et al. [49] observed that phenolic compounds in peat inhibited the activity of extracellular enzymes (β-glucosidase, phosphatase, sulphatase, chitinase, and xylosidase) by 14–32%.

The peak heights (2983 + 2921 and 1713) are characteristic of C–H and C=O and were used to describe the hydrophilic and hydrophobic components of the peat soil under different land use changes. The hydrophobic component (C–H) for the peat samples obtained in this study is less than those found by Šimon et al. [46] for mineral soil but less than those found by Heller et al. [28] for the undrained and drained bog. The low hydrophobic components (C–H) of the peat soil exhibited by forest plantation in the 0–10 cm indicate the decomposition of more labile carbohydrates than the grassland and the cutaway bog. The land use change did not influence the hydrophilic of the peat soil found in this study. The results of our study suggest that the composition of soil organic matter is primarily dominated by hydrophobic organic constituents, in contrast to the anticipated dominance of hydrophilic constituents, which agrees with the results observed by Laudicina et al. [50] and Šimon et al. [46] on mineral soil. The grassland exhibited reduced levels of aromatic compounds and aromaticity compared to the forest and the cutaway bog. This observation can be attributed to the higher decomposition rate of lignin and phenolic components within the organic matter of peat soil.

Low hydrophobicity of the studied land use was observed, which may be attributed to the enhanced microbial activity under aerobic conditions, which may increase the content of C=O groups and the subsequent formation of stable microbial carbohydrates [44]. According to Lachacz et al. [51], the hydrophobicity in soils rich in organic matter depends on the SOM quality (e.g., microbial biomass, hydrophilic functional group, etc.) and irreversible organic matter drying. Kalisz et al. [17] made a similar observation when studying the effect of peat drainage on labile organic matter and water repellency/hydrophobicity in NE Poland. Their findings revealed that converting undisturbed bog to grassland caused a lower hydrophobicity, which they attributed the lower grassland's hydrophobicity to drainage effects and microbial activity expressed as hot water-extractable carbon. The forest plantation exhibited higher hydrophobicity than the grassland due to the additional organic matter input from the litter falls and plant debris, as well as the deep root system of the plants. According to Kalbitz et al. [52], forest floor litter contributes to the aromatic and lignin-derived compounds of organic matter, which explain the higher hydrophobicity exhibited by the forest plantation than the grassland.

Moreover, the grassland exhibited the highest humification indices due to their intensive management practices (grazing, fertiliser). Humification is the process by which relatively less resistant organic compounds transform, forming more stable and/or less biodegradable organic complexes. These complexes, known as humus, play a crucial role in soil's overall formation of humic substances [53,54]. One of the most important management practices is applying lime and fertilisers (e.g., manure, NPK fertiliser) to neutralise the peat soil's natural acidity, which provides a favourable environment for microorganisms to biodegradation organic matter. Additionally, the least humified peat originating from the grassland may be assigned to the decline of the carbohydrate component, carboxylate and carboxylic acid compounds, indicating a higher degree of oxidation, and lower soil acidity, favouring microbial decomposition of the peat [10]. The degree of decomposition was higher in the cutaway bog and the grassland than in the forest plantation, which may probably be assigned to more favourable degradation conditions and faster SOM cycling stimulated by the enhanced aerobic decomposition of the phenolics and lignins than the forest plantation [29,55].

## 5 Conclusion

The research evaluates carbon chemistry signatures, components, and characteristics of organic matter of peat soil affected by land use change in temperate peat soil. The carbon chemistry signatures of the SOM, such as aliphatic methyl and methylene, C=O stretching of amide groups, aromatic C=C, strong H-bond C=O of conjugated ketones and O–H deformation and C– O stretching of phenolics, secondary alcohols and the C–O stretching of polysaccharides were affected by land use change especially when converting natural peatland to grassland. The hydrophobicity and the aromaticity follow the order active bog > forest plantation > industrial cutaway bog > grassland. The grassland peat sample and cutaway bog recorded a higher degree of decomposition due to the drastic decrease in the recalcitrant and labile organic matter fractions. The humification indices were higher in the grassland. In addition, the aromatic and the aromaticity nearly exhibited a positive correlation with all the carbon chemistry signatures found in peat soil. At the same time, the degree of degradation was negatively correlated with hydrophilic functional groups and hydrophobicity.

Based on our current findings, it could be acknowledged that converting peatland to the studied land use through drainage negatively altered peat soil's recalcitrant and labile organic matter fractions, which may affect the globally important process such as carbon sequestration. Therefore, to limit further transformation and degradation of SOM from the studied land use, especially the grassland, ditches blocking should be implemented in combination with rewetting to increase the water table, which in turn decreases the microbial decomposition of the recalcitrant and labile organic matter fractions.

## Funding

Not applicable.

## Author contribution statement

Apori Samuel Obeng; Michelle Giltrap; Furong Tian: Conceived and designed the experiments; Performed the experiments; Analyzed and interpreted the data; Contributed reagents, materials, analysis tools or data; Wrote the paper.

Julie Dunne: Conceived and designed the experiments; Performed the experiments; Analyzed and interpreted the data; Wrote the paper.

## Data availability statement

Data will be made available on request.

## Additional information

No additional information is available for this paper.

## Declaration of competing interest

The authors declare that they have no known competing financial interests or personal relationships that could have appeared to influence the work reported in this paper.

## References

[1] Pendall E., Bridgham S., Hanson P.J., Hungate B., Kicklighter D.W., Johnson D.W., Law B.E., Luo Y., Megonigal J.P., Olsrud M. (2004). Below-ground process responses to elevated CO2 and temperature: a discussion of observations, measurement methods, and models. New Phytol..

[2] Thormann M.N. (2006). Diversity and function of fungi in peatlands: a carbon cycling perspective. Can. J. Soil Sci..

[3] Limpens J., Berendse F., Blodau C., Canadell J.G., Freeman C., Holden J., Roulet N., Rydin H., Schaepman-Strub G. (2008). Peatlands and the carbon cycle: from local processes to global implications–a synthesis. Biogeosciences.

[4] Urbanová Z., Bárta J. (2016). Effects of long-term drainage on microbial community composition vary between peatland types. Soil Biol. Biochem..

[5] Apori S.O., Mcmillan D., Giltrap M., Tian F. (2022).

[6] Alcántara V., Don A., Well R., Nieder R. (2016). Deep ploughing increases agricultural soil organic matter stocks. Global Change Biol..

[7] Swails E., Jaye D., Verchot L., Hergoualc’h K., Schirrmann M., Borchard N., Wahyuni N., Lawrence D. (2018). Will CO2 emissions from drained tropical peatlands decline over time? Links between soil organic matter quality, nutrients, and C mineralisation rates. Ecosystems.

[8] Broder T., Blodau C., Biester H., Knorr K.-H. (2012). Peat decomposition records in three pristine ombrotrophic bogs in southern Patagonia. Biogeosciences.

[9] Heller C., Zeitz J. (2012). Stability of soil organic matter in two northeastern German fen soils: the influence of site and soil development. J. Soils Sediments.

[10] Chapman S.J., Campbell C.D., Fraser A.R., Puri G. (2001). FTIR spectroscopy of peat in and bordering Scots pine woodland: relationship with chemical and biological properties. Soil Biol. Biochem..

[11] Sahrawat K.L. (2004). Organic matter accumulation in submerged soils. Adv. Agron..

[12] Artz R.R., Chapman S.J., Robertson A.J., Potts J.M., Laggoun-Défarge F., Gogo S., Comont L., Disnar J.-R., Francez A.-J. (2008). FTIR spectroscopy can be used as a screening tool for organic matter quality in regenerating cutover peatlands. Soil Biol. Biochem..

[13] Schaumann G.E., Bertmer M. (2008). Do water molecules bridge soil organic matter molecule segments?. Eur. J. Soil Sci..

[14] Angst G., Mueller K.E., Nierop K.G., Simpson M.J. (2021). Plant-or microbial-derived? A review on the molecular composition of stabilised soil organic matter. Soil Biol. Biochem..

[15] Szajdak L., Szatylowicz J. (2010). Impact of drainage on hydrophobicity of fen peat-moorsh soils. Mires Peat.

[16] Tang R., Clark J.M., Bond T., Graham N., Hughes D., Freeman C. (2013). Assessment of potential climate change impacts on peatland dissolved organic carbon release and drinking water treatment from laboratory experiments. Environ. Pollut..

[17] Kalisz B., Lachacz A., Glazewski R. (2015). Effects of peat drainage on labile organic carbon and water repellency in NE Poland. Turk. J. Agric. For..

[18] Min K., Freeman C., Kang H., Choi S.-U. (2015). The regulation by phenolic compounds of soil organic matter dynamics under a changing environment. BioMed Res. Int..

[19] Wu Y., Zhang N., Slater G., Waddington J.M., de Lannoy C.-F. (2020). Hydrophobicity of peat soils: characterisation of organic compound changes associated with heat-induced water repellency. Sci. Total Environ..

[20] Dungait J.A., Hopkins D.W., Gregory A.S., Whitmore A.P. (2012). Soil organic matter turnover is governed by accessibility not recalcitrance. Global Change Biol..

[21] Capriel P., Beck T., Borchert H., Gronholz J., Zachmann G. (1995). Hydrophobicity of the organic matter in arable soils. Soil Biol. Biochem..

[22] Utami S.N.H., Suswati D. (2016). Chemical and spectroscopy of peat from West and Central Kalimantan, Indonesia in relation to peat properties. Int J Env. Agric Res..

[23] Franco C.M.M., Clarke P.J., Tate M.E., Oades J.M. (2000). Hydrophobic properties and chemical characterisation of natural water repellent materials in Australian sands. J. Hydrol..

[24] Ellerbrock R.H., Gerke H.H., Bachmann J., Goebel M.-O. (2005). Composition of organic matter fractions for explaining wettability of three forest soils. Soil Sci. Soc. Am. J..

[25] Hewelke E., Szaty\lowicz J., Gnatowski T., Oleszczuk R. (2016). Effects of soil water repellency on moisture patterns in a degraded sapric histosol. Land Degrad. Dev..

[26] Moore P.A., Lukenbach M.C., Kettridge N., Petrone R.M., Devito K.J., Waddington J.M. (2017). Peatland water repellency: importance of soil water content, moss species, and burn severity. J. Hydrol..

[27] Ibarra J., Munoz E., Moliner R. (1996). FTIR study of the evolution of coal structure during the coalification process. Org. Geochem..

[28] Heller C., Ellerbrock R.H., Roßkopf N., Klingenfuß C., Zeitz J. (2015). Soil organic matter characterisation of temperate peatland soil with FTIR-spectroscopy: effects of mire type and drainage intensity. Eur. J. Soil Sci..

[29] Zaccheo P., Cabassi G., Ricca G., Crippa L. (2002). Decomposition of organic residues in soil: experimental technique and spectroscopic approach. Org. Geochem..

[30] Cocozza C., D’orazio V., Miano T.M., Shotyk W. (2003). Characterization of solid and aqueous phases of a peat bog profile using molecular fluorescence spectroscopy, ESR and FT-IR, and comparison with physical properties. Org. Geochem..

[31] Billes F., Mohammed-Ziegler I. (2007). Vibrational spectroscopy of phenols and phenolic polymers. Theory, experiment, and applications. Appl. Spectrosc. Rev..

[32] Chakraborty J., Das S. (2017). Application of spectroscopic techniques for monitoring microbial diversity and bioremediation. Appl. Spectrosc. Rev..

[33] Niemeyer J., Chen Y., Bollag J.-M. (1992). Characterisation of humic acids, composts, and peat by diffuse reflectance Fourier-transform infrared spectroscopy. Soil Sci. Soc. Am. J..

[34] Simkovic I., Dlapa P., Doerr S.H., Mataix-Solera J., Sasinkova V. (2008). Thermal destruction of soil water repellency and associated changes to soil organic matter as observed by FTIR spectroscopy. Catena.

[35] Šimon T., Javuurek M., Mikanova O., Vach M. (2009). The influence of tillage systems on soil organic matter and soil hydrophobicity. Soil Tillage Res..

[36] Pärnpuu S., Astover A., Tõnutare T., Penu P., Kauer K. (2022). Soil organic matter qualification with FTIR spectroscopy under different soil types in Estonia. Geoderma Reg.

[37] Matějková Š., Šimon T. (2012). Application of FTIR spectroscopy for evaluation of hydrophobic/hydrophilic organic components in arable soil. Plant Soil Environ..

[38] Rezanezhad F., Price J.S., Quinton W.L., Lennartz B., Milojevic T., Van Cappellen P. (2016). Structure of peat soils and implications for water storage, flow and solute transport: a review update for geochemists. Chem. Geol..

[39] Hammond R.F., Brennan L.E. (2003).

[40] Creamer R., O'Sullivan L. (2018).

[41] Gondar D., Lopez R., Fiol S., Antelo J.M., Arce F. (2005). Characterisation and acid–base properties of fulvic and humic acids isolated from two horizons of an ombrotrophic peat bog. Geoderma.

[42] Grube M., Lin J.-G., Lee P.H., Kokorevicha S. (2006). Evaluation of sewage sludge-based compost by FT-IR spectroscopy. Geoderma.

[43] Ellerbrock R.H., Gerke H.H., Böhm C. (2009). In situ DRIFT characterisation of organic matter composition on soil structural surfaces. Soil Sci. Soc. Am. J..

[44] Chefetz B., Chen Y., Hadar Y. (1998). Purification and characterisation of laccase from Chaetomium thermophilium and its role in humification. Appl. Environ. Microbiol..

[45] Negassa W., Acksel A., Eckhardt K.-U., Regier T., Leinweber P. (2019). Soil organic matter characteristics in drained and rewetted peatlands of northern Germany: chemical and spectroscopic analyses. Geoderma.

[46] Mudgil D., Barak S., Khatkar B.S. (2012). X-ray diffraction, IR spectroscopy and thermal characterisation of partially hydrolysed guar gum. Int. J. Biol. Macromol..

[47] Zaccone C., Miano T.M., Shotyk W. (2007). Qualitative comparison between raw peat and related humic acids in an ombrotrophic bog profile. Org. Geochem..

[48] Olk D.C., Brunetti G., Senesi N. (2000). Decrease in humification of organic matter with intensified lowland rice cropping a wet chemical and spectroscopic investigation. Soil Sci. Soc. Am. J..

[49] Freeman C., Fenner N., Ostle N.J., Kang H., Dowrick D.J., Reynolds B., Lock M.A., Sleep D., Hughes S., Hudson J. (2004). Export of dissolved organic carbon from peatlands under elevated carbon dioxide levels. Nature.

[50] Laudicina V.A., Novara A., Barbera V., Egli M., Badalucco L. (2015). Long-term tillage and cropping system effects on chemical and biochemical characteristics of soil organic matter in a Mediterranean semiarid environment. Land Degrad. Dev..

[51] Lachacz A., Nitkiewicz M., Kalisz B. (2009). Water repellency of post-boggy soils with a various content of organic matter. Biologia (Bratisl.)..

[52] Kalbitz K., Meyer A., Yang R., Gerstberger P. (2007). Response of dissolved organic matter in the forest floor to long-term manipulation of litter and throughfall inputs. Biogeochemistry.

[53] Hayes M.H., Swift R.S. (2020). Vindication of humic substances as a key component of organic matter in soil and water. Adv. Agron..

[54] Senesi N., Loffredo E. (2018). Soil Phys. Chem..

[55] Guo X., Du W., Wang X., Yang Z. (2013). Degradation and structure change of humic acids corresponding to water decline in Zoige peatland, Qinghai-Tibet Plateau. Sci. Total Environ..

